# FAK activates AKT-mTOR signaling to promote the growth and progression of MMTV-Wnt1-driven basal-like mammary tumors

**DOI:** 10.1186/s13058-020-01298-3

**Published:** 2020-06-03

**Authors:** Ritama Paul, Ming Luo, Xueying Mo, Jason Lu, Syn Kok Yeo, Jun-Lin Guan

**Affiliations:** 1 Department of Cancer Biology, University of Cincinnati College of Medicine, Cincinnati, OH 45267 USA; 2 Department of Internal Medicine, Division of Hematology and Oncology, University of Michigan, Ann Arbor, MI 48109 USA; 3 Division of Biomedical Informatics, Cincinnati Children’s Hospital Research Foundation, Cincinnati, OH 45229 USA

**Keywords:** FAK, WNT1, Basal-like breast cancer, mTOR

## Abstract

**Background:**

Breast cancer is a heterogeneous disease. Hence, stratification of patients based on the subtype of breast cancer is key to its successful treatment. Among all the breast cancer subtypes, basal-like breast cancer is the most aggressive subtype with limited treatment options. Interestingly, we found focal adhesion kinase (FAK), a cytoplasmic tyrosine kinase, is highly overexpressed and activated in basal-like breast cancer.

**Methods:**

To understand the role of FAK in this subtype, we generated mice with conditional deletion of FAK and a knock-in mutation in its kinase domain in MMTV-Wnt1-driven basal-like mammary tumors. Tumor initiation, growth, and metastasis were characterized for these mice cohorts. Immunohistochemical and transcriptomic analysis of Wnt1-driven tumors were also performed to elucidate the mechanisms underlying FAK-dependent phenotypes. Pharmacological inhibition of FAK and mTOR in human basal-like breast cancer cell lines was also tested.

**Results:**

We found that in the absence of FAK or its kinase function, growth and metastasis of the tumors were significantly suppressed. Furthermore, immunohistochemical analyses of cleaved caspase 3 revealed that loss of FAK results in increased tumor cell apoptosis. To further investigate the mechanism by which FAK regulates survival of the Wnt1-driven tumor cells, we prepared an isogenic pair of mammary tumor cells with and without FAK and found that FAK ablation increased their sensitivity to ER stress-induced cell death, as well as reduced tumor cell migration and tumor sphere formation. Comparative transcriptomic profiling of the pair of tumor cells and gene set enrichment analysis suggested mTOR pathway to be downregulated upon loss of FAK. Immunoblot analyses further confirmed that absence of FAK results in reduction of AKT and downstream mTOR pathways. We also found that inhibition of FAK and mTOR pathways both induces apoptosis, indicating the importance of these pathways in regulating cell survival.

**Conclusions:**

In summary, our studies show that in a basal-like tumor model, FAK is required for survival of the tumor cells and can serve as a potential therapeutic target.

## Background

Breast cancer is the second most common cause of cancer death in women across the world. Most of these deaths occur due to metastatic spread and disease relapse. Breast cancer is a heterogeneous disease and transcriptome profiling has led to the identification of at least six molecular subtypes of the disease, including luminal A, luminal B, Her2-enriched, basal-like, claudin-low, and normal-like [1]. Importantly, each of these subtypes is associated with varying prognoses and has differential sensitivities to conventional therapies. For example, the basal and claudin-low subtypes are known to have poorest outcomes in patients as compared to the other subtypes but do not respond to hormonal or HER2-targeted therapy [2]. This highlights the necessity of finding novel treatment strategies to target basal-like breast cancers, since effective treatment options are still lacking.

Wnt proteins are a family of ligands that bind to Frizzled receptors and initiate an intracellular signaling pathway leading to activation of various genes related to developmental pathways. The canonical Wnt pathway involves the stabilization of β-catenin, which can enter the nucleus and transactivate Wnt target genes [3]. This canonical Wnt pathway has been implicated in human basal-like breast tumors. Wnt-1 (initially named as int-1), a member of the family of Wnt ligands, was the first protooncogene identified to be activated by the nearby insertion of mouse mammary tumor virus (MMTV) proviruses in mammary tumors of infected mice [4]. Transgenic expression of Wnt1 using MMTV-LTR enhancer is sufficient to form mammary adenocarcinomas. The MMTV-Wnt1 mouse model develops mammary tumors which can be predominantly classified as the basal subtype. These tumors are enriched for mammary stem cells (MaSC) and predominantly express basal cell markers [5]. Transcriptomic profile of these tumors resembles a MaSC-like signature [6]. Hence, MMTV-Wnt1 serves as a basal-like breast cancer mice model to study the relevance of various signaling pathways active in human basal-like breast cancer.

Focal adhesion kinase (FAK) is a cytoplasmic tyrosine kinase that is activated downstream of integrin receptors in response to binding to the extracellular matrix [7]. Other than ECM receptors, FAK also plays a role as a mediator of cell signaling downstream of growth factor and cytokine receptors. FAK is highly expressed in a number of cancers including breast, intestinal, and ovarian, in which it is known to promote cancer growth and metastasis through both kinase-dependent and independent mechanisms [8]. Previous studies have shown that FAK plays an important role in promoting mammary tumor development and progression in mouse models of luminal B [9–12] and HER-2 [13] subtypes of breast cancer. Additionally, FAK is highly expressed in triple-negative and basal-like breast cancer [14, 15]. A recent study showed that FAK also contributed to the malignancy of a human triple-negative breast cancer cell line MDA-MB-231 [16]. However, direct evidence for the in vivo importance of FAK in basal-like breast cancer is still lacking. Hence, we evaluated the effect of deletion of FAK and disruption of its kinase function in a basal-like breast cancer model.

FAK has been shown to control tumor cell survival, proliferation, and migration through both kinase-dependent and independent pathways. FAK can directly phosphorylate Src family kinases and form a heterodimer complex with Src [17], which leads to the phosphorylation of p130Cas adaptor molecule and activation of multiple downstream signaling events, including the RAS-Erk pathway in PyMT induced mammary tumors [18]. Reduction in cyclin D1 levels in PyMT tumors has been associated with reduction in tumor cell proliferation upon loss of FAK [9]. Kinase function of FAK has also been shown to activate the PI3K-Akt pathway, which can protect cells from apoptosis and promote survival [8]. FAK can also promote survival through its kinase-independent scaffolding function in the nucleus by promoting MDM2-mediated degradation of p53 [19].

One of the direct consequences of PI3K-AKT pathway is downstream activation of mTOR signaling. mTOR is a large protein kinase associated with different protein partners to form two independently regulated hetero-oligomeric complexes, the rapamycin-sensitive and rapamycin-insensitive mTOR complex (mTORC) 1 and 2, respectively [20]. AKT inhibits TSC2 by phosphorylating it, leading to activation of Rheb-GTPase which then directly binds and activates mTORC1. mTORC2 on the other hand can be directly activated by AKT [21]. mTORC1 can activate protein translation and rewire cellular metabolism, thereby promoting cell growth and survival. It is highly activated in a wide variety of cancers [22]. Thus, FAK could also affect the mTOR pathway through its activation of PI3K-AKT signaling. Indeed, a recent study showed that FAK inhibition reduced mTOR activation in MCF7 and MDA-MB-231 cells [23]. Our lab has also previously found that FAK directly interacts with TSC2 and promotes S6 kinase phosphorylation [24]. Because FAK can activate a number of downstream effectors, it is important to study the contributions of these various pathways in mediating FAK regulation of basal-like breast cancer.

In this study, we demonstrate that though FAK is dispensable for the onset of Wnt1-driven mammary tumors, disruption of FAK’s kinase activity suppressed Wnt1-driven tumor growth and progression through compromised tumor cell survival. We found that FAK activates the AKT-mTOR pathway in these tumors, which in turn supports the survival of the tumor cells. In summary, our studies show that in a basal-like mammary tumor model, FAK is required for survival of the tumor cells and could potentially serve as a therapeutic target in the treatment of basal-like breast cancer.

## Methods

### Mice

FAK Ctrl (FAK f/f), cKO (FAK f/f, MMTV-Cre), and cKD (FAK f/KD, MMTV-Cre) transgenic mice have been described previously [9, 25–27]. MMTV-CRE Line F mice were obtained from NCI [28]. MMTV-Wnt1 mice were obtained from Dr. Yi Li [29] and were crossed with FAK Ctrl, cKD, and cKO mice. We previously showed that the MMTV-Cre targets both the luminal and the basal compartment of the mammary glands [9, 27, 30]. Our group generated the kinase defective knock-in allele of FAK (KD) by mutating K454 to R in the catalytic domain of FAK (in exon 16 of FAK genomic DNA), and these were initially described in [26]. Mice were palpated every 7 days after weaning, and the size of tumors was measured with a caliper and recorded. Mice were housed and handled according to local, state, and federal regulations, and all experimental procedures were carried out according to the guidelines of the Institutional Animal Care and Use Committee at the University of Cincinnati.

### Reagents and antibodies

Antibodies used in this study include FAK (CST-3285), phospho-FAK Y397 (CST 8556), GAPDH (CST 2118), Ki67 (Spring Bioscience m3062), cleaved caspase 3 (CST 9661), CD31 (Dianovo, DIA310), CD8 (Invitrogen MA1-80231), Vinculin (Sigma V4505), phospho-4EBP1Ser65 (CST 9451), phospho-4EBP1Thr37/46 (CST 2855), 4EBP1 (CST 9644), p-S6K (CST 9205), S6K (Santa Cruz SC-230), phospho-AKT Ser473(CST 4060), phospho-AKT Thr308 (CST 5473), and AKT (CST9272). Inhibitors used in this study include PF-562271 (Cayman), PP242 (MedChem Express), thapsigargin (Cayman), and tunicamycin (Cayman).

### Cell culture, treatments, transfection, and transduction of cells

Tumor cells derived from FAK f/f Wnt1 tumors were cultured in DMEM/F12 supplemented with 10% FBS, 10 ng/ml EGF, 20 μg/ml insulin, and 50 units/ml penicillin-streptomycin. Lentivirus production and transduction of the tumor cells with Cre-ERT were carried out as described previously [31]. Transfection experiments were carried out using Lipofectamine 2000 Reagent (Invitrogen). Deletion of FAK was induced by culturing with 100 nM 4-hydroxy-tamoxifen. Amino acid starvation of the tumor cells was carried out by culturing them for 48 h in HBSS. MDA_MB231, CAL1851, and HCC186 were cultured in DMEM medium supplemented with 10% FBS and 50 units/ml penicillin-streptomycin. Viability of cells after treatment was determined using Alamar Blue reagent (Thermo Fisher).

### Migration, wound healing, and sphere formation assay

Cells were seeded at a density of 25,000 cells/well in Boyden chambers coated with growth factor reduced Matrigel (BD Biosciences) and incubated for 24 h. Cells on the membrane were then fixed with ice cold ethanol and stained with crystal violet. Cells which have invaded to the lower side of the membrane were then quantified. For wound healing assay, 10,000 tumor cells were plated per well in a 96-well plate. Wounds were made with Woundmaker (Essen Biosciences) and imaged using Incucyte (Essen Biosciences). Quantifications were done using ImageJ software. For sphere formation assay, tumor cells were plated at density of 10,000 cells/ml in a 96-well low attachment plate and cultured in MEBM medium supplemented with 0.2% B-27, 20 ng/ml EGF, 5 μg/ml insulin, 20 μg/ml Gentamycin, and 0.5 μg/ml Hydrocortisone for 7–10 days.

### Flow cytometry

Unattached dead cells and attached cells after treatment were collected after brief trypsinization and stained using BD Pharmingen AnnexinV apoptosis detection kit as per manufacturer’s protocol. Stained cells were analyzed using FACSAria. Flow cytometry data were analyzed using FlowJo software.

### METABRIC dataset analysis

METABRIC dataset [32] was downloaded from cBioportal [33]. PTK2 gene was queried on cBioportal, and K-M survival plot was visualized and downloaded. Gene amplification and mRNA expression data was analyzed using GraphPad Prism and MS Excel.

### RNA sequencing of tumor cells

RNA sequencing experiments were performed by the Genomics, Epigenomics and Sequencing Core in University of Cincinnati. Briefly, RNA from tumor cells was isolated using mirVana miRNA Isolation Kit (Thermo Scientific) according to the manufacturer’s instructions for total RNA isolation. Targeted RNA enrichment was achieved using NEBNext Poly(A) mRNA Magnetic Isolation Module (New England BioLabs) and PrepX mRNA Library kit (WaferGen) combined with Apollo 324 NGS automated library prep system was used for library preparation. Cluster generation and HiSeq sequencing were carried out using the cBot and HiSeq systems (Illumina) respectively. To analyze differential gene expression, sequence reads were aligned to the genome using standard Illumina sequence analysis pipeline, which was analyzed by The Laboratory for Statistical Genomics and Systems Biology in the University of Cincinnati. The RNA-seq data have been deposited in the GEO database under accession code GSE146659.

### Mammary gland whole mounts, histology, and immunohistochemistry

Fourth abdominal mammary glands were excised at 5 weeks after birth, and whole mounts stained with carmine alum were analyzed, as described previously [27]. Mammary tumors or lungs were harvested from mice and subjected to analysis by histology, immunohistochemistry, as described previously [9, 34]. Briefly, tumors were fixed overnight in 10% phosphate-buffered 10% formalin (Fisher Scientific), dehydrated in alcohol gradients, xylene, and paraffin before being embedded. Next, they were sectioned into 5-μm-thick slices. Unstained tissue sections were first deparaffinized in xylene (3 times, 5 min each), rehydrated in graded ethanol solutions (100, 95, 70%, 50%, and 30%), and stained with H&E. For immunohistochemistry, tumor sections were first subjected to antigen retrieval (sodium citrate buffer, pH − 6.8), stained with different antibodies and mounted with Permount mounting medium (Fisher chemicals). For quantification of metastatic nodules, we obtained three sections from each lung tissue that were 200 μM apart. All three sections were stained with H&E and scanned through a light microscope at × 4 magnification. A number of metastatic nodules were identified based on histology and counted.

### Immunoblotting

Lysates were prepared using modified RIPA buffer as described previously [27] with the addition of Halt protease and phosphatase inhibitors (Thermo Scientific). Protein concentrations were then quantified by the BCA method, subjected to SDS-PAGE, and analyzed by immunoblotting as described previously [27].

### Statistics

Data were plotted as means ± SEM, and statistical significance was determined using a two-tailed *t* test, one-way ANOVA, or two-way ANOVA followed by Tukey’s multiple comparison test wherever applicable. For tumor-free survival curves, statistical significance was determined using a log-rank test (Mantel-Cox). The threshold for significance of *p* values was 0.05.

## Results

### Elevated FAK expression is associated with worse patient prognosis and is more prevalent in basal-like breast cancers

**Fig. 1:**
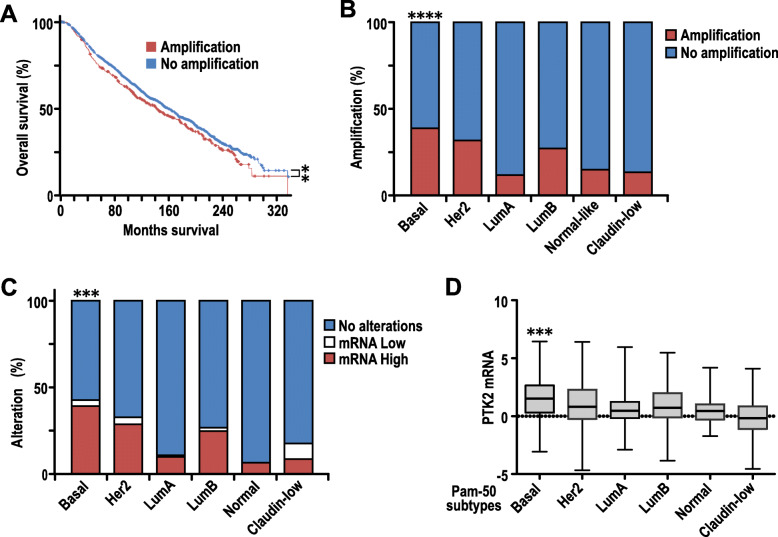
Elevated FAK expression is associated with worse patient prognosis and are more prevalent in basal-like breast cancers. **a** Kaplan–Meier survival plot from analysis of the METABRIC dataset of overall survival curve of breast cancer patients with or without FAK gene amplification. Log-rank test, **p < 0.01. **b** Analysis of percentage of patients with FAK gene amplification across all prediction analysis of microarray 50 (PAM50) classified breast cancer subtypes in METABRIC dataset. Fisher’s exact test, ****p (basal vs other subtypes) < 0.0001. **c** Analysis of percentage of patients with high FAK mRNA across all PAM50 classified breast cancer subtypes in METABRIC dataset. Chi-square test, ****p (basal vs other subtypes) < 0.0001. **d** Boxplot representation of FAK mRNA expression levels in the PAM50 subtypes. One-way ANOVA, basal-like versus other subtypes, ***p < 0.001

To establish potential associations corresponding to the expression of FAK in human breast cancers, we queried the METABRIC dataset [32] that includes a large patient cohort (2509 tumors) through cBioPortal [33] and found that FAK gene (Official Symbol: PTK2) was amplified in 415 (21%) of these tumors. Importantly, patients with amplified FAK had a significantly poorer prognosis (median overall survival = 139.5 months) relative to patients without FAK amplifications (median overall survival = 164.3 months, log-rank test *p* value = 0.001577) (Fig. 1a). Intriguingly, when the tumors were stratified according to PAM50 subtypes [35], there was a further enrichment of FAK amplifications (38.7%) in patients with basal-like breast cancer (Fisher’s exact test, *p* < 0.0001) (Fig. 1b). In agreement with FAK amplification status, a higher proportion of basal-like breast cancers in this cohort had higher levels of FAK mRNA expression (mRNA *z*-score threshold = ± 2, for high and low respectively) (chi-square test, *p* < 0.0001) (Fig. 1c), and basal-like breast cancers exhibited the highest mean log_2_(fold change) of 1.414 (Fig 1d). Collectively, these associations suggested that FAK may play a more prominent role in human basal-like breast cancers and highlights the necessity of studying its in vivo relevance in a basal-like pre-clinical model.

### Disruption of FAK kinase function suppresses the growth and progression of MMTV-Wnt1-driven mammary tumors

**Fig. 2:**
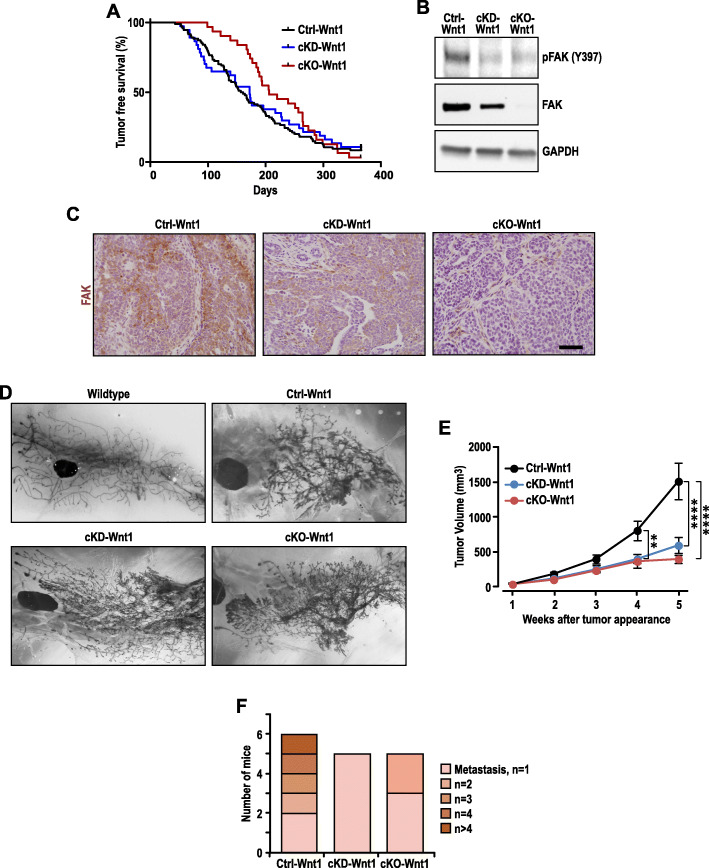
FAK deletion suppresses tumor growth and progression in MMTV-Wnt1-driven basal-like mammary tumors. **a** Tumor-free survival curves of Ctrl-Wnt1 (*n* = 94), cKO-Wnt1 (*n* = 37), and cKD-Wnt1 (*n* = 31) mice. **b** Immunoblots showing levels of phosphorylated FAK, total FAK, and GAPDH in Ctrl-Wnt1, cKD-Wnt1, and cKO-Wnt1 tumors (*n* = 3 for each sample). **c** Immunohistochemistry staining of FAK in Ctrl-Wnt1, cKD-Wnt1, and cKO-Wnt1 tumor sections. **d** Whole mounts of mammary glands from 5-week-old wildtype, Ctrl-Wnt1, cKD-Wnt1, and cKO-Wnt1 females. **e** Tumor growth curves of Ctrl-Wnt1(*n* = 22), cKO-Wnt1 (*n* = 10), and cKD-Wnt1 (*n* = 22) mice. Two-way ANOVA followed by Tukey’s multiple comparisons test, **p ≤ 0.01, ****p ≤ 0.0001. **f** Bar chart showing number of mice (out of *n* = 21 for each genotype) with various number of metastatic nodules in their lungs quantified from H&E-stained lung sections prepared when mice have similar primary tumor size, chi-square test, *p* (Ctrl-Wnt1 vs cKD-Wnt1) = 0.02, *p* (Ctrl-Wnt1 vs cKO-Wnt1) = 0.03

The MMTV-Wnt1 mouse model spontaneously develops mammary tumors which can be predominantly classified as the basal subtype [6]. Hence, we employed this model to study the importance of FAK in basal-like breast cancer. To determine the role of FAK and its kinase function in MMTV-Wnt1-driven basal-like mammary tumors, FAK floxed allele (FAK^fl^) [25, 9] and FAK kinase dead knock-in allele (FAK^KD^) [26, 27] were crossed with MMTV-Wnt1 and MMTV-Cre mice which express the Cre recombinase in mammary epithelial cells. From these, three cohorts of female mice with the genotypes FAK^fl/fl^, MMTV-Cre, and MMTV-Wnt1 (designated as cKO-Wnt1); FAK^fl/KD^, MMTV-Cre, and MMTV-Wnt1 (designated as cKD-Wnt1); and FAK^fl/KD^ and MMTV-Wnt1, along with FAK^fl/fl^ and MMTV-Wnt1 (collectively designated as Ctrl-Wnt1) were established. These mice were then examined for the appearance of tumors, in order to gauge the importance of FAK (conditionally deleted in mammary tumor cells of cKO-Wnt1 mice) and its kinase activity (only kinase-defective protein encoded by the KD allele was expressed whereas the floxed allele deleted in mammary tumor cells of cKD-Wnt1 mice) for mammary tumor development and progression. In the Ctrl-Wnt1 cohort, palpable tumors can be detected at a median latency of 159 days (Fig. 2a), which is similar to previous reports [4, 29]. cKD-Wnt1 mice developed tumors with a slightly increased median latency at 172 days and an overall very similar time course as Ctrl-Wnt1 mice, suggesting that FAK kinase activity was not required for mammary tumor development in this mouse model. Deletion of FAK in cKO-Wnt1 mice showed a greater trend of delay in tumor appearance at a median latency of 206 days, but the differences between cKO-Wnt1 and Ctrl-Wnt1 mice was not statistically significant (log-rank test, *p* = 0.1246).

Next, we prepared lysates from these tumors and analyzed by immunoblotting to verify the deletion of FAK alleles by MMTV-Cre to ablate FAK expression or its kinase activity in cKO-Wnt1 and cKD-Wnt1 mice, respectively. As expected, the level of phosphorylated FAK (pY397) was diminished in tumors derived from cKD-Wnt1 and cKO-Wnt1 mice relative to tumors from Ctrl-Wnt mice (Fig. 2b). Furthermore, FAK protein expression was completely ablated in cKO-Wnt1 tumors and reduced in cKD-Wnt1 tumors (expression of the kinase-defective FAK from the KD allele). The decrease and ablation in FAK protein levels in cKD-Wnt1 and cKO-Wnt1 tumors, respectively, relative to Ctrl-Wnt1 tumors were further validated by immunohistochemical analysis of tumor sections from these mice (Fig. 2c).

To explore a potential basis for the apparently lack of a role for FAK in mammary tumor development in MMTV-Wnt1 model, we examined mammary gland whole mounts of these mice at an earlier age, as one characteristic feature of this model is Wnt-driven mammary ductal hyperbranching that is accompanied by abnormal alveolar formation in nulliparous mice [4]. Consistent with previous reports, we found that while wildtype mice (i.e., without Wnt1 overexpression) had clearly defined primary branches with minimal side-branching or alveolar formation, mammary glands in Ctrl-Wnt1 showed dense side branches and diffuse alveolar hyperplasia at 5 weeks of age (Fig. 2d). Interestingly, during this developmental time point, cKD-Wnt1 and cKO-Wnt1 glands were indistinguishable from Ctrl-Wnt1 glands (Fig. 2d) indicating that FAK and its kinase function might not contribute significantly to the aberrant developmental phenotypes induced by Wnt1. This lack of any effect on the early expansion of Wnt1-responsive cells upon FAK ablation or loss of its kinase activity could potentially account for at least in part the apparently similar tumor development for the three cohorts of mice Ctrl-Wnt1, cKD-Wnt1, and cKO-Wnt1 (see Fig 2a).

We next monitored tumor growth following the appearance of tumors in these mice by caliper measurements weekly. In contrast to the initial mammary tumor development, cKD-Wnt1 and cKO-Wnt1 tumors showed significantly reduced growth rates relative to Ctrl-Wnt1 tumors (Fig. 2e). Since the loss of FAK or its kinase function led to a similar suppression in tumor growth, these results support that the importance of FAK kinase activity in the promotion of mammary tumor growth driven by MMTV-Wnt1. At 5 weeks after the initial detection of primary mammary tumors, histological analysis of lung sections showed metastatic nodules in about 28.5% (6 out of 21) of Ctrl-Wnt1 mice (Figs. S1A and S1C). At the same time points, cKD-Wnt1 and cKO-Wnt1 mice had much smaller primary tumor volumes (see Fig. 2e). Thus, we examined lung sections of these mice at later time points when primary tumors reached similar size as that of Ctrl-Wnt1 tumors in 5 weeks to alleviate differences caused by varying primary tumor burden. At these time points, we detected lung metastatic nodules in about 23.8% (5 out of 21) of cKD-Wnt1 and cKO-Wnt1 mice (Fig. S1A). Although this decrease in the fraction of mice with metastasis was not statistically significant, we noted that the number of nodules per lung section was decreased in cKD-Wnt1 and cKO-Wnt1 relative to Ctrl-Wnt1 (Fig. 2f). Whereas Ctrl-Wnt1 mice had a range of 1 to more than 4 nodules per section (most had more than 2 metastatic nodules), cKD-Wnt1 and cKO-Wnt1 mice had only 1 or 2 metastatic nodules per section (chi-square test, *p* (Ctrl-Wnt1 vs cKD-Wnt1) = 0.02, *p* (Ctrl-Wnt1 vs cKO-Wnt1) = 0.03). However, we did not note any significant changes in the area of the metastatic nodules (Fig. S1B). This suggests that though FAK reduces the number of metastatic events, it might not be affecting the growth of the metastatic cells at the secondary organ. Together, these results suggest that although FAK is dispensable for the onset of Wnt1-driven mammary tumors, disruption of FAK’s kinase activity suppressed Wnt1-driven tumor growth and metastasis.

### Disruption of FAK kinase function increases apoptosis in MMTV-Wnt1-driven mammary tumors

**Fig. 3:**
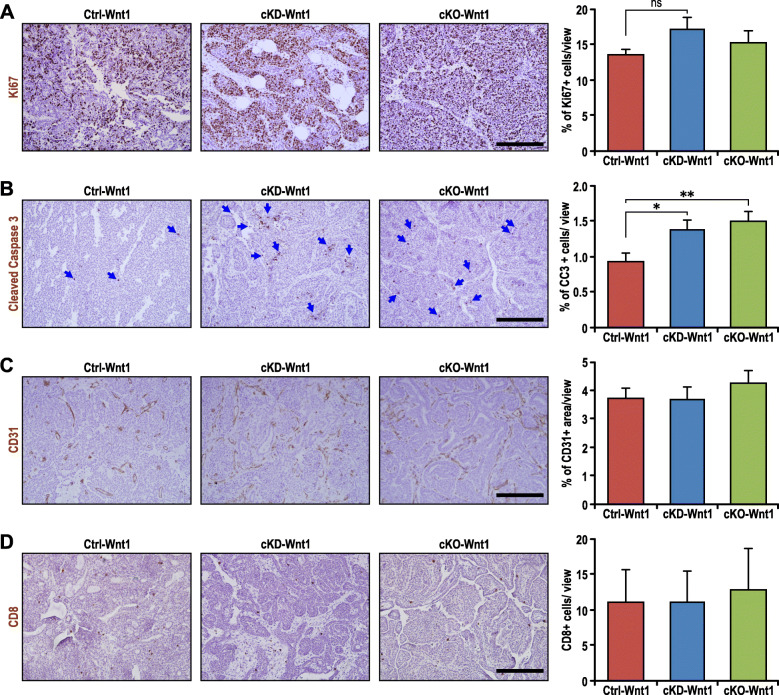
Loss of FAK kinase function reduces survival of MMTV-Wnt1-driven basal-like mammary tumors. **a** Representative images of Ctrl-Wnt1, cKD-Wnt1, and cKO-Wnt tumors immunostained for Ki67 and corresponding quantification of percentage of positive cells per field of view (*n* = 4 for each genotype). **b** Representative images of Ctrl-Wnt1, cKD-Wnt1, and cKO-Wnt tumors immunostained for cleaved caspase 3 and corresponding quantification of percentage of positive cells per field of view (Ctrl-Wnt1, cKD-Wnt *n* = 8; cKO-Wnt *n* = 7). **c** Representative images of Ctrl-Wnt1, cKD-Wnt1, and cKO-Wnt tumors immunostained for CD31 and corresponding quantification of percentage of CD31-positive area per field of view (*n* = 3). **d** Representative images of Ctrl-Wnt1, cKD-Wnt1, and cKO-Wnt tumors immunostained for CD8 and corresponding quantification of percentage of CD8-positive cells per field of view (Ctrl-Wnt1 *n* = 7; cKD-Wnt, cKO-Wnt *n* = 5). All statistical tests for **c**–**f** include ANOVA followed by Tukey’s multiple comparisons test, *p < 0.05, **p ≤ 0.01

Consequently, we went on to determine the basis for the suppression of tumor growth in both the cKD-Wnt1 and cKO-Wnt1 cohorts. The proliferative and apoptotic rates in these tumors were also examined by Ki67 and cleaved caspase-3 immunostaining respectively (Figs. 3a, b). Although there were no significant differences in the percentage of Ki67 positive cells between Ctrl-Wnt1, cKD-Wnt1, and cKO-Wnt1 tumors (Fig. 3a), an evident increase in the percentage of cleaved caspase 3-positive cells can be detected in cKD-Wnt1 and cKO-Wnt1 tumors relative to Ctrl-Wnt1 tumors (Fig. 3b). Inspection of the percentage of CD31^+^ areas (Fig. 3c) and CD8^+^ T-cells (Fig. 3d) within these tumors also revealed no significant differences, thus excluding the possibility that the differences observed in tumor growth (see Fig. 2e) were due to defects in angiogenesis or cytotoxic T cell infiltration. Hence, these results indicated that loss of FAK kinase function in Wnt1-driven tumors led to a cell-autonomous defect in survival and increased apoptosis.

### FAK ablation sensitizes Wnt1-driven tumor cells to ER stress-induced cell death and reduces their activities in migration and sphere formation

**Fig. 4:**
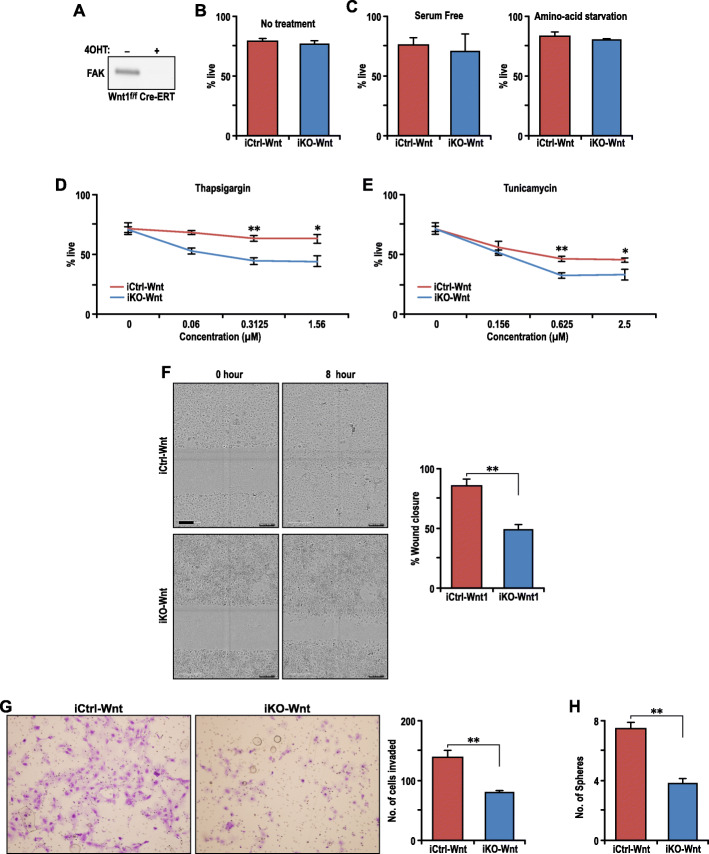
FAK ablation sensitizes Wnt1-driven tumor cell to ER stress-induced cell death and reduces their activities in migration and sphere formation. **a** Immunoblot showing level of FAK in iCtrl-Wnt and iKO-Wnt cells. **b**, **c**. Bar graph quantification from AnnexinV-PI flow cytometry of iCtrl-Wnt and iKO-Wnt cells after 48 h of no treatment (**b**) and serum starvation or amino-acid starvation (**c**), *n* = 6. Triplicates from two independent experiments. **d**, **e** Dose response curves from AnnexinV-PI flow cytometry of iCtrl-Wnt and iKO-Wnt cells treated for 48 h with thapsigargin (**d**) or tunicamycin (**e**). *n* = 6. Duplicates from three independent experiments. Student’s *t* test, *p ≤ 0.05, **p ≤ 0.01. **f** Representative image of scratch-wound assay of iCtrl-Wnt and iKO-Wnt cells at 0 and 8 h and corresponding quantification of percentage of wound closure, *n* = 6, Student’s *t* test, *p* = 0.003. **g** Representative image of iCtrl-Wnt and iKO-Wnt cells migrated in Matrigel-coated transwell assay and corresponding quantification of cells invaded, *n* = 6, Student’s *t* test, *p* = 0.002). **h** Bar graph representation of number of spheres formed per 1000 iCtrl-Wnt and iKO-Wnt cells under no attachment condition, *n* = 24, 12 wells from two independent repeats, Student’s *t* test, *p* < 0.01

To further examine the role of FAK in Wnt1-driven mammary tumor growth and metastasis, spontaneously immortalized mammary tumor cells from Ctrl-Wnt1 (i.e., MMTV-Wnt1; Fak^fl/fl^) mice were prepared, and then transduced with recombinant retroviruses encoding Cre-ERT [31, 36]. This enables the activation of Cre-recombinase upon administration of 4-OHT, leading to deletion of FAK in isogenic Wnt1-driven mammary tumor cells. Deletion of FAK was confirmed by immunoblot of lysates from these isogenic pair of cells that were treated with either vehicle control (designated as iCtrl-Wnt cells) or 4-OHT (designated as iKO-Wnt cells) (Fig. 4a). Surprisingly, however, we did not detect any difference in cell survival between iCtrl-Wnt and iKO-Wnt cells under standard culture conditions (Fig. 4b), despite increased apoptosis of mammary tumor cells in cKO-Wnt1 mice, suggesting that FAK deletion may affect cell survival only under stress conditions in vivo. To explore this possibility, we examined cell survival of these cells under a number of stress conditions in culture. We found that FAK deletion did not change survival of Wnt1-driven mammary tumor cells under stress conditions including serum or amino-acid starvation (Fig. 4b). Interestingly, treatment with the endoplasmic reticulum (ER) stress inducer, thapsigargin, reduced the survival of iKO-Wnt cells, but not iCtrl-Wnt cells, under the same conditions (Fig. 4d). Thapsigargin inhibits ER calcium ATPase and hence blocks the transport of calcium from cytoplasm to ER, resulting in depletion of ER calcium stores. To distinguish whether FAK deletion caused increased sensitivity towards ER stress induction or depletion of calcium stores in particular, we used another ER stress inducer, tunicamycin, which functions independent of ER calcium channels, in similar assays. Consistent with our observations with thapsigargin, tunicamycin reduced survival of iKO-Wnt cells to a greater extent relative to iCtrl-Wnt cells (Fig. 4e). We also stained the tumors for phospho-EIF2α, a marker of ER stress response, and did not find any differences in its levels between Ctrl-Wnt1, cKD-Wnt1, and cKO-Wnt1 tumors (Fig. S2). Altogether, these results indicate that the integrated stress response was induced to a similar level in tumor cells with or without FAK, but in the absence of FAK, the tumor cells have a lower threshold for tolerance to ER stress.

Besides cell survival, we also examined the effect of FAK deletion on a number of other cellular activities in Wnt1-driven mammary tumor cells. We found that iKO-Wnt cells were less migratory as compared to iCtrl-Wnt cells as observed in the wound healing assay (Fig. 4f). Similarly, invasive activity of iKO-Wnt cells was also decreased relative to iCtrl-Wnt cells as measured by Matrigel-coated transwell migration assays (Fig. 4g). Lastly, we explored if FAK deletion affects tumor sphere formation under non-adherent conditions. We detected a reduction of tumor sphere formation by iKO-Wnt cells compared to iCtrl-Wnt cells (Fig. 4h). These results suggest an important role of FAK in the regulation of migration and sphere formation of Wnt tumor cells, and decreases in these cellular functions upon FAK deletion could also contribute to their reduced mammary tumor growth and/or metastasis in vivo. The increased sensitivity to ER stress-induced cell death also suggests a potential combinatorial therapeutic strategy of FAK ablation along with ER stress induction to treat Wnt1-driven mammary tumors.

### Transcriptomic analysis reveals reduced AKT-mTORC1 signaling upon FAK ablation

**Fig. 5:**
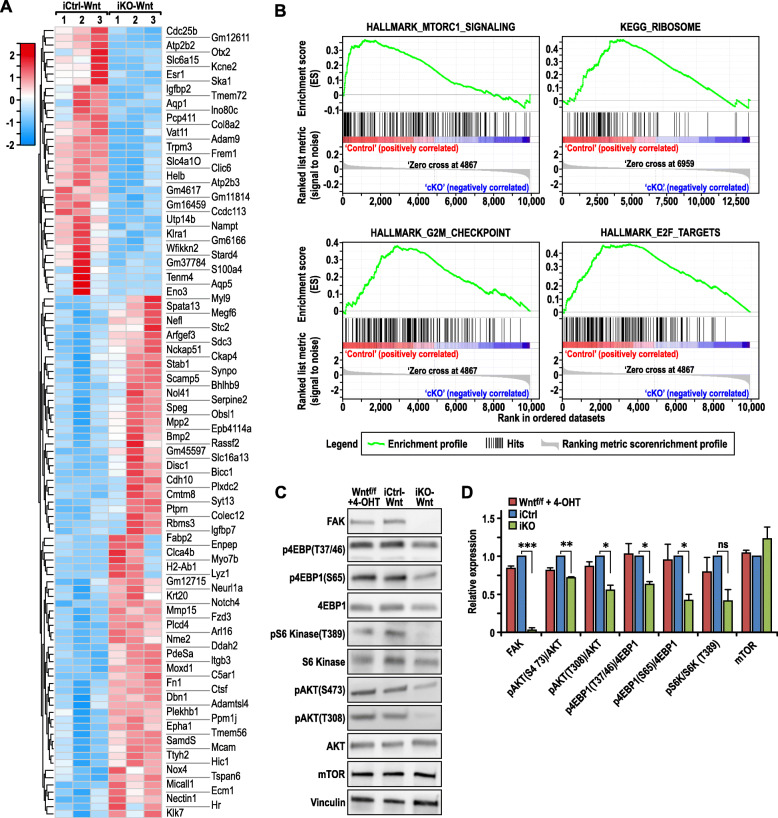
Transcriptomic analysis indicate mTORC1 pathway is affected upon loss of FAK. **a** Heat map from RNA sequencing data showing the expression levels of top genes (*p* < 0.01) that are changed in triplicates of iCtrl vs iKO tumor cells (red—high, blue—low). **b** Gene set enrichment analysis plot (GSEA) showing ribosome gene signature, mTORC1 signaling gene signature, G2M checkpoint, and E2F target gene signature to be enriched in iCtrl cells, FDR < 0.1. **c** Immunoblot of FAK, AKT, and mTOR substrates and their phosphorylation in control FAKf/f; MMTV-Wnt1 tumor cells (Wntf/f) treated with 4OHT, iCtrl-Wnt, and iKO-Wnt cells that had been starved for 2 h in serum free media. **d** Quantification of immunoblot in **c**, one-way ANOVA, *p ≤ 0.05, **p ≤ 0.01, ***p < 0.001

To gain further insights into potential mechanisms by which FAK regulates Wnt1-driven mammary tumors, we examined FAK deletion-induced changes in various cellular processes and signaling pathways in an unbiased manner by transcriptomic analysis. Since independent tumors from cKO-Wnt1 mice may acquire mutations that activate divergent pathways respectively, we employed the isogenic pair of iCtrl-Wnt and iKO-Wnt cells for these studies which limits the confounding factor of other mutations in individual tumors. Three independent replicates of mRNA samples were prepared from iCtrl-Wnt and iKO-Wnt cells, reverse-transcribed and subjected to transcriptomic analysis (RNA sequencing). A total of 109 genes were found to be differentially expressed between iCtrl-Wnt and iKO-Wnt cells from this analysis using a threshold of *p* < 0.01 (Fig. 5a). We then performed gene set enrichment analysis (GSEA) using this dataset to identify the cellular processes and signaling pathways that were likely to be perturbed based on the differences in gene expression between these two cells. This analysis revealed that among the differentially expressed genes, genes related to four signaling pathways and cellular processes, including mTORC1 signaling, ribosome biogenesis, G2-M checkpoint, and E2F targets, were significantly enriched (Hallmark and KEGG gene sets with FDR < 0.1) and also suggested to have decreased activities in iKO-Wnt compared to iCtrl-Wnt tumor cells (Fig. 5b). Although we detected upregulation for multiple genes (see Fig. 5a), GSEA analysis did not show any increased signaling pathways or biological processes in iKO-Wnt tumor cells.

It is worth noting that mTORC1 plays a crucial role in the regulation of translation, which is dependent on ribosome biogenesis; thus, we focused on the mTORC1 pathway in subsequent analysis. To rule out a possibility that the mTOR pathway was affected due to 4OHT treatment, Ctrl-Wnt1 cells that were not transduced with Cre-ERT were treated with 4OHT and our results indicated that the mTOR pathway was not affected by 4OHT treatment when compared with iCtrl-Wnt cells (Fig. 5c). Accordingly, the activity of mTORC1 was inspected in the iCtrl-Wnt and iKO-Wnt cells by determining the levels of phosphorylated 4EBP1 and S6 Kinase, two main mTOR substrates (Fig. 5c, d). An apparent decrease in the levels of phosphorylated 4EBP1 can be observed both at Serine 65 and Threonine 37/46 residues in cells that lacked FAK (Fig. 5c, d). Levels of pS6 Kinase was also found to be reduced in iKO-Wnt cells relative to iCtrl-Wnt cells, although the reduction did not reach statistical significance (Fig. 5c, d). In addition, we also checked phosphorylation of AKT, an upstream activator of mTOR. We observed AKT phosphorylation was decreased both at Serine 473 and Threonine 308 sites in iKO-Wnt cells. The P13K-PDK pathway mainly regulates the phosphorylation of AKT at Threonine 308 whereas Serine 473 phosphorylation of AKT is mainly regulated by mTORC2 [37]. This illustrates the possibility of regulation of both P13K and mTORC2 activity by FAK. Additionally, we did not observe any difference in mTOR levels, indicating that FAK affects mTOR activity but not the expression of mTOR. As a whole, these results suggest that FAK is required to activate AKT-mTOR signaling in Wnt1-driven tumors.

### Inhibition of mTOR pathway leads to apoptosis of the Wnt tumor cells and human basal-like breast cancer cells

**Fig. 6:**
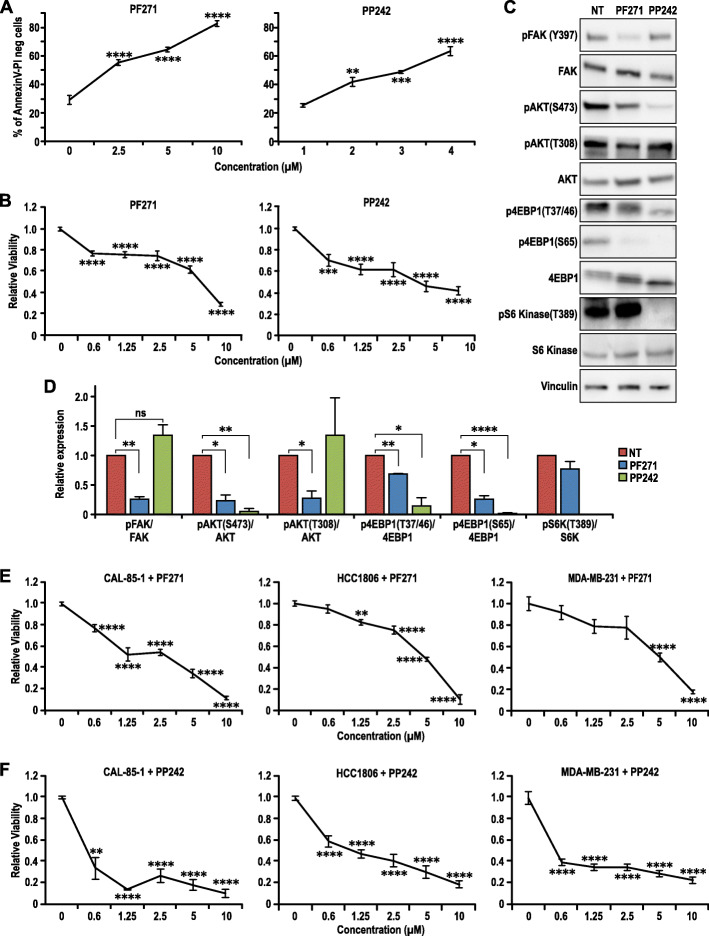
Inhibition of FAK and mTOR leads to apoptosis of the Wnt tumor cells and human basal-like breast cancer cells. **a** Percentage of Annexin V-positive iCtrl-Wnt cells treated with increasing doses of PF271 and PP242 for 48 h *n* = 6, duplicates from three independent repeats. **b** Dose response curves from Alamar Blue assay of iCtrl-Wnt cells treated with PF271 and PP242 for 72 h, *n* = 9, triplicates from three independent repeats. **c** Immunoblot showing inhibition of FAK and mTOR pathway in iCtrl-Wnt cells upon treatment with PF271(5uM) and PP242 (2.5uM). **d** Quantification of immunoblot in **c**. **e** Dose response curves of MDA-MB-231, CAL-85-1, and HCC1806 cells treated with PF271 for 72 h. *n* = 9, duplicates from three independent repeats. **f** Dose response curves of MDA-MB-231, CAL-85-1, and HCC1806 cells treated with PP242 for 72 h. *n* = 9, duplicates from three independent repeats. All statistical tests include ANOVA followed by Tukey’s multiple comparisons test, *p < 0.05, **p ≤ 0.01, ***p < 0.001, ****p ≤ 0.0001

As loss of FAK-induced apoptosis in the mammary tumors of cKO-Wnt1 and cKD-Wnt1 mice and FAK deletion in Wnt tumor cells in vitro resulted in reduction of the mTOR signaling pathway, we wanted to explore whether mTOR pathway is responsible for apoptosis of the Wnt tumor cells. We inhibited the mTOR pathway with PP242 and FAK with PF-562271 (PF271) in the iCtrl-Wnt cells. As expected and consistent with genetic ablation and loss of kinase activity (i.e., in cKD-Wnt1 mice), FAK inhibition by PF271 increased apoptosis of iCtrl-Wnt cells in a dose-dependent manner (Fig. 6a). Moreover, measurements of cell survival by Alamar Blue assay confirmed that FAK inhibition decreased cell viability accordingly (Fig. 6b). Similar experiments showed that mTOR inhibition by PP242 also increased apoptosis and reduced cell survival (Fig. 6a, b). We also treated the iKO-Wnt cells with PF271 and PP242 and observed that they were less sensitive to both these drugs (Fig. S3). Since the iKO cells no longer express FAK, it is expected that they will not be sensitive to FAK inhibition. Also, since the mTOR pathway is downregulated in the iKO cells, they might be less dependent on this pathway, hence less sensitive to mTOR inhibition. Overall, the results suggested that reduced mTOR signaling is responsible at least in part for the cell death phenotype upon FAK deletion.

In further support of this hypothesis and as observed for FAK deletion in iKO-Wnt cells (see Fig. 5c), FAK inhibition by PF271 reduced mTOR activity as measured by phosphorylation of 4EBP1 (T37/46, S65) and AKT (S473) (Fig. 6c, d), downstream targets of mTORC1 and mTORC2, respectively. Lastly, we examined the effect of inhibition of FAK and mTOR on human basal-like breast cancer cell lines, including CAL-85-1, HCC1806, and MDA-MB-231 cells. We found that treatment of all three cells by either PF271 or PP242 led to reduced cell viability with increasing doses (Fig. 6e, f). Together, these results suggest that FAK regulation of mTOR signaling pathway contributes to its promotion of tumor growth and metastasis of Wnt1-driven and basal-like breast cancer.

## Discussion

Basal-like breast cancer is an aggressive subtype of breast cancer with limited treatment options [2]. It has been reported that FAK protein level and its phosphorylation are highly elevated in triple-negative breast cancers [14, 15], which include basal-like subtype. Consistent with these studies, we found a greater proportion of basal-like tumors as compared to any other subtypes in the METBRIC dataset, to contain genetic FAK amplification and/or high levels of FAK mRNA. To study the mechanistic relevance of these associations in vivo, we knocked out FAK and disrupted its kinase function through a knock-in mutation, in the Wnt1-induced basal-like breast cancer mice model. We found that indeed FAK plays a prominent role in the growth of these tumors. Previously, we and others showed that FAK deletion in MMTV-PyMT tumors, classified as luminal B subtype, suppressed tumor growth [9], and our studies also showed defective maintenance of mammary cancer stem cells in the mutant mice [27]. Other studies showed that FAK plays an important role in ERBB2 (HER2+ subtype)-induced mammary tumorigenesis [13]. Our results provide direct evidence of the importance of FAK in a basal-like breast cancer model and complement previous studies to support FAK as a potential therapeutic target for all major subtypes of human breast cancer.

While FAK promotes mammary tumor growth and metastasis in all models of different subtypes, there were several notable differences between our findings in mouse model for basal-like breast cancer subtype and previous studies in models of other subtypes. As in other models and consistent with a role for FAK in regulating cell migration in many cell types [9, 25], we found reduced cell migration upon FAK deletion in Wnt1-driven mammary tumor cells. In addition, we also found reduced tumor sphere formation in Wnt1-driven mammary tumor cells upon loss of FAK, as in PyMT-driven tumor cells we reported earlier [9]. However, FAK ablation decreased the proliferation of mammary tumor cells derived from some other mouse models of breast cancer [13, 27, 38]. FAK deletion in Wnt1-driven mammary tumor did not change tumor cell proliferation but increased apoptosis which likely also contributes to the reduced tumor growth and metastasis in this model. The other surprising finding is a lack of effect on mammary tumor development upon FAK deletion in this model, despite the apparently significant inhibition of tumor growth and metastasis after tumor appearance. It is possible that the increased apoptosis following FAK deletion or the loss of its kinase activity only occurs when mammary tumors reach palpable size (i.e., tumor appearance as defined in our assays, see Fig. 2) in cKO-Wnt1 and cKD-Wnt1 mice. Interestingly, we only detected reduced survival of iKO-Wnt cells vs iCtrl-Wnt cells under ER stress conditions, but not normal culture conditions, in vitro. Such stress conditions (ER stress or potentially other stresses) may only exist when the tumor reach palpable size in vivo, which could explain an effect of FAK deletion in tumor growth and metastasis at later stage due to increased apoptosis. Interestingly, we found a modest difference in tumor initiation times between the cKO and cKD tumors. Previously, we have reported that the kinase function of FAK regulates luminal progenitors [27]. However, mammary stem cells (MaSCs) were regulated by a kinase independent function and not dependent on the kinase function of FAK. In the MMTV-Wnt1 model, expansion of MaSCs has been well documented. Thus, the loss of the kinase-independent function of FAK in cKO-Wnt1 mice, but not cKD-Wnt1 or Ctrl-Wnt1, which could compromise the ability of MMTV-Wnt1 induced expansion of MaSCs, may explain the trend for the delayed tumor initiation times in cKO-Wnt1 tumors relative to cKD-Wnt1 and Ctrl-Wnt1 tumors.

Interestingly, there is a large change in cell viability upon treatment with FAK inhibitor (Fig. 6b), as compared to when FAK is ablated in vivo (see Fig. 3d). This could be due to differences between acute inhibition of FAK and constitutive deletion of FAK. For the tumors in vivo, the cells have coped with FAK ablation for long periods of time (since pre-neoplastic stages) and thus may exhibit less dependence on FAK. The doses of FAK inhibitor used in these studies were appropriate for in vitro treatments and had been used as per previous studies [39, 40].

Gunther et al. showed that Wnt1-initiated mammary tumors require Wnt signaling for tumor maintenance and the tumors regress if Wnt signaling is blocked [41]. Previously, FAK has been reported to promote Wnt signaling in colorectal cancer by phosphorylating GSK3β thereby blocking the degradation of β-catenin and its accumulation [42]. However, we did not find any changes in β-catenin levels upon blocking FAK signaling (data not shown). This illustrates the possibility of requirement of independent signaling pathways activated by FAK other than the Wnt pathway in maintaining tumor growth. Based on an unbiased transcriptomic analysis of isogenic Wnt1-driven mammary tumor cells with or without FAK, we found that mTORC1 signaling, ribosome biogenesis, G2-M checkpoint, and E2F target-related genes were significantly downregulated in the absence of FAK. E2F has been previously reported to be an upstream activator of mTOR [43]. In addition, mTOR has been indicated to control multiple steps in ribosome biogenesis [44]. These studies further indicate that mTOR and its related pathways might be downregulated as a result of FAK deletion. We further showed that loss of FAK disrupts phosphorylation of AKT at Serine 473 and Threonine 308. Phosphorylation of AKT at Serine 473 and Threonine 308 is usually mediated by mTORC2 and PDK1 respectively. Previously, we have shown that FAK binds PI3K and regulates its activity [45]. Hence, our results indicate that AKT is regulated downstream of FAK through both the PI3K-PDK1 and mTORC2 cascade. In addition, we found decreased mTORC1 signaling, which is consistent with the reduction in AKT activity and the data from our transcriptomic analysis. The mTOR signaling pathway plays a critical role in mediating growth stimulatory pathways in Wnt tumors [46]. Indeed, we found that mTOR inhibitor PP242 targeting both mTORC1 and mTORC2 could induce apoptosis in Wnt1-driven mammary tumor cells, supporting a role for mTOR to at least partially mediate FAK regulation of tumor growth and metastasis.

Our studies also showed that in the absence of FAK, the tumor cells were sensitive to ER stress-inducing agents. A recent study suggested that FAK protects endothelial cells from ER stress-induced mitochondrial damage and cell death by activating STAT3 [47]. This raises the possibility that the absence of FAK confers a survival vulnerability of the tumor cells to induction of ER stress. Whether FAK can directly trigger phosphorylation of STAT3 or if there are other mediators involved in this process needs to be investigated in order to gain more mechanistic insights about the role of FAK in mediating cell survival under ER stress. Thus, we have unraveled a potential combinatorial therapeutic strategy of FAK ablation along with induction of ER stress in Wnt1-driven tumor cells.

## Conclusions

In summary, we show that FAK plays an important role in the maintenance of tumor cell survival in Wnt1-driven basal-like mammary tumors and can serve as a potential therapeutic target for the treatment of basal-like breast cancer.

## Supplementary information

**Additional file 1: Fig. S1.** a. Bar chart showing percentage of mice with metastasis. b. Quantification of area of metastatic nodule normalized to area of the lung. c. Representative images from H&E staining of metastatic nodules in the lung. **Fig. S2.** a. Immunohistochemistry for phosphoEIF2α of tumor sections from the three genotypes. b. Quantification of a. **Fig. S3.** a. Dose response curves from Alamar Blue assay of iCtrl-Wnt (Red) and iKO-Wnt cells (Blue) treated with PF271 and PP242 for 72 h, *n* = 9, triplicates from three independent repeats. One-way ANOVA (iCtrl-Wnt vs iKO-Wnt), ** denotes *p* < 0.01, **** denotes *p* ≤ 0.0001.

## Data Availability

The datasets used and/or analyzed during the current study are available from the corresponding author on reasonable request.
