# Correction to: FAK activates AKT-mTOR signaling to promote the growth and progression of MMTV-Wnt1-driven basal-like mammary tumors

**DOI:** 10.1186/s13058-020-01310-w

**Published:** 2020-06-29

**Authors:** Ritama Paul, Ming Luo, Xueying Mo, Jason Lu, Syn Kok Yeo, Jun-Lin Guan

**Affiliations:** 1 Department of Cancer Biology, University of Cincinnati College of Medicine, Cincinnati, OH 45267 USA; 2 Department of Internal Medicine, Division of Hematology and Oncology, University of Michigan, Ann Arbor, MI 48109 USA; 3 Division of Biomedical Informatics, Cincinnati Children’s Hospital Research Foundation, Cincinnati, OH 45229 USA

**Correction to: Breast Cancer Res (2020) 22:59**

**https://doi.org/10.1186/s13058-020-01298-3**

Following publication of the original article [1], the authors identified an error in Figure 6. The labels of “% of AnnexinV-PI negative cells” should be corrected to “% of AnnexinV-PI positive cells”.

The correct Figure is given below.

The original article has been corrected.


