# Supplementary figures and images for: FAK activates AKT-mTOR signaling to promote the growth and progression of MMTV-Wnt1-driven basal-like mammary tumors
Source: Breast Cancer Res. 2020 Jun 3;22:59. doi: 10.1186/s13058-020-01298-3 (PMC7268629; doi:10.1186/s13058-020-01298-3)

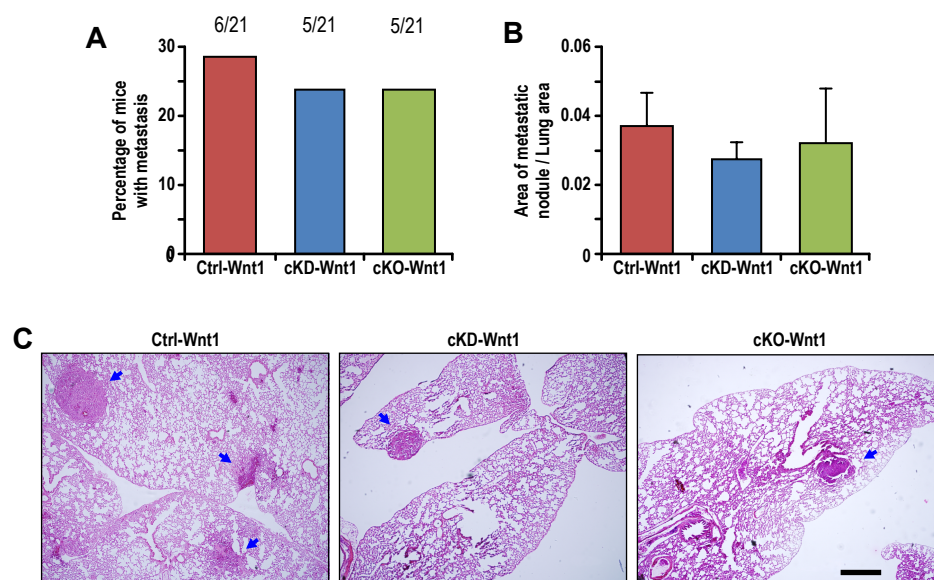

Figure S1 (1-26-20)

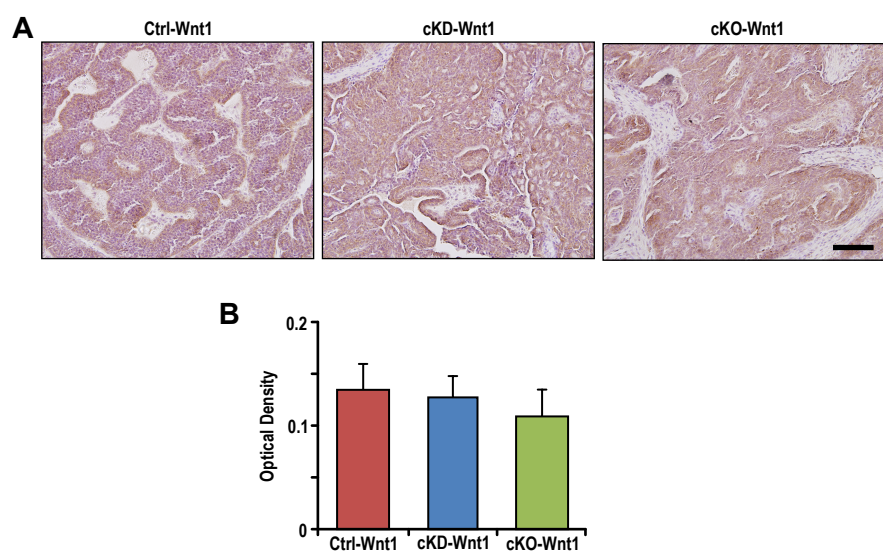

Figure S2 (1-26-20)

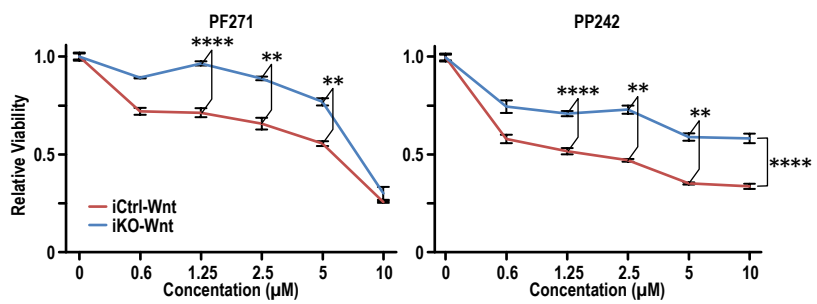

Figure S3 (1-26-20)

Supplement: Supplementary file 1 — Additional file 1: Fig. S1. a. Bar chart showing percentage of mice with metastasis. b. Quantification of area of metastatic nodule normalized to area of the lung. c. Representative images from H&E staining of metastatic nodules in the lung. Fig. S2. a. Immunohistochemistry for phosphoEIF2α of tumor sections from the three genotypes. b. Quantification of a. Fig. S3. a. Dose response curves from Alamar Blue assay of iCtrl-Wnt (Red) and iKO-Wnt cells (Blue) treated with PF271 and PP242 for 72 h, n = 9, triplicates from three independent repeats. One-way ANOVA (iCtrl-Wnt vs iKO-Wnt), ** denotes p < 0.01, **** denotes p ≤ 0.0001. [file 13058_2020_1298_MOESM1_ESM.pdf]
